# Evaluation of the Postprandial-Hyperglycemia-Suppressing Effects and Safety of Short-Term Intake of Mulberry Leaf and Water Chestnut Tea: A Randomized Double-Blind Placebo-Controlled Crossover Trial

**DOI:** 10.3390/nu17142308

**Published:** 2025-07-13

**Authors:** Yuya Shinkawa, Midori Yasuda, Yuichiro Nishida, Mikiko Tokiya, Yusuke Takagi, Akiko Matsumoto, Atsushi Kawaguchi, Megumi Hara

**Affiliations:** 1 Department of Medical Science, Graduate School of Medical Science, Saga University, Saga 849-8501, Japan; 2 Department of Health and Nutrition Sciences, Nishikyushu University, Saga 842-8585, Japan; 3 Department of Preventive Medicine, Faculty of Medicine, Saga University, Saga 849-8501, Japan; 4 Department of Social and Environmental Medicine, Faculty of Medicine, Saga University, Saga 849-8501, Japan; 5 Education and Research Center for Community Medicine, Faculty of Medicine, Saga University, Saga 849-8501, Japan

**Keywords:** continuous glucose monitoring, FreeStyle Libre, diabetes, crossover study, mulberry leaf and water chestnut tea

## Abstract

**Background/Objectives**: Postprandial hyperglycemia is a risk factor for diabetes and cardiovascular diseases, even in healthy individuals. Kanzaki mulberry leaf and water chestnut tea (MW tea), a blend of mulberry (*Morus alba*) leaves and water chestnut (*Trapa japonica*) leaves and husks, is rich in polyphenols and 1-deoxynojirimycin (DNJ) and may suppress postprandial glucose spikes, but evidence regarding its short-term daily intake is limited. This study aimed to evaluate the postprandial glycemic response and safety of two-week MW tea consumption using continuous glucose monitoring (CGM). **Methods**: We conducted a randomized, double-blind, placebo-controlled, two-period crossover trial involving 31 participants. Each intervention period lasted two weeks, separated by a one-week washout. Participants consumed either MW tea or a placebo before meals. Interstitial glucose levels were measured every 15 min using CGM. Postprandial glucose responses were recorded every 15 min for 180 min after a standardized meal on the first day of each period. The primary outcome was the coefficient of variation (CV) in glucose levels, calculated using data from the central 10 days of each intervention period. Safety was assessed using CGM-derived hypoglycemia metrics and blood test results. **Results**: The CV of glucose levels during the MW tea period was significantly lower than during the placebo period (mean difference: 0.02, *p* = 0.0006). A significant reduction in 1 h postprandial glucose area under the curve was also observed. No significant differences were found in hypoglycemia occurrence, liver/renal/inflammatory markers, or self-reported adverse symptoms. Notably, 1,5-anhydroglucitol (1,5-AG) levels significantly increased during MW tea intake, suggesting improved glycemic control. **Conclusions**: Short-term consumption of Kanzaki MW tea effectively suppressed postprandial glucose variability without safety concerns. These findings support MW tea as a promising natural supplement for glycemic management and the prevention of diabetes.

## 1. Introduction

In recent years, postprandial blood glucose elevation has been increasingly recognized as a risk factor for chronic diseases such as diabetes and cardiovascular diseases [1]. Postprandial hyperglycemia, even in healthy individuals, is known to cause vascular endothelial dysfunction in the short term and elevate the risk of diabetes and cardiovascular events in the long term [2]. Therefore, managing postprandial glucose levels is not only important for diabetes prevention but may also offer broader public health benefits.

In addition to pharmacological interventions, dietary approaches and the use of naturally derived foods and beverages have garnered attention as methods for managing postprandial blood glucose levels. Recent studies have suggested that polyphenols and other compounds with α-glucosidase inhibitory activities may help suppress postprandial glycemic spikes [3,4]. Consequently, the development of functional foods and beverages containing components that suppress glucose absorption is in progress.

One such example is the “Kanzaki mulberry leaf and water chestnut tea” developed in Kanzaki City. This beverage is prepared by blending powdered mulberry leaves and water chestnut husk. The mulberry leaves are derived from *Morus alba* (family Moraceae), and the water chestnut husk from *Trapa japonica* (family Lythraceae), both of which are edible plants native to East Asia and have been investigated for their potential glycemic effects [5]. The husk of water chestnut is rich in polyphenols, which reportedly inhibits carbohydrate-digesting enzymes and reduces postprandial glycemic response in both mice and humans [3,4]. Specifically, the husk of the water chestnut contains eugeniin, 1,2,3,6-tetra-*O*-galloyl-d-glucopyranose (TGG), and trapain, which are hydrolyzed polyphenols [5]. Mulberry leaves contain 1-deoxynojirimycin (DNJ), which suppresses postprandial blood glucose elevation [6,7]. DNJ is classified as a polyhydroxylated piperidine alkaloid, which acts as a potent inhibitor of α-glucosidase and has been studied for its therapeutic potential in glycemic control [8,9,10]. Furthermore, a synergistic effect on enzyme inhibition was observed when mulberry and water chestnut components were combined [5].

However, most previous studies focused on the effects of a single intake, and there is limited evidence on the impact of continued daily consumption as part of a typical diet. Data on the safety of sustained intake, such as the risk of hypoglycemia or gastrointestinal symptoms, are scarce. To evaluate the effects of Kanzaki mulberry leaf and water chestnut tea in real-life contexts, it is essential to examine their impact on short-term continuous intake.

This study aimed to evaluate the postprandial glycemic response and safety of short-term daily consumption of Kanzaki mulberry leaf and water chestnut tea. A notable feature of this study is the use of continuous glucose monitoring (CGM) (FreeStyle Libre, Abbott), which enables detailed observation of postprandial glucose fluctuations. This approach allows for a more realistic assessment than traditional indices such as fasting glucose or single postprandial measurements. Furthermore, a crossover design was employed to minimize inter-individual variability. Daily lifestyle factors that potentially influence glycemic responses, such as physical activity, sleep duration, and meal content, were also measured in detail to improve the precision of the evaluation.

## 2. Materials and Methods

### 2.1. Participants

The participants were adults who expressed interest in monitoring their own blood glucose levels. Eligible individuals were selected through prescreening tests and questionnaires, excluding those who met the following criteria: (1) fasting blood glucose ≥ 126 mg/dL or glycated hemoglobin (HbA1c) ≥ 6.5%, indicating a diagnosis of diabetes, (2) under continuous medical treatment with medication, (3) regular consumption of pharmaceuticals, foods for specified health uses, foods with functional claims, or health supplements may affect the study outcomes, (4) history or presence of serious conditions involving the heart, liver, kidneys, or digestive system, (5) anemia (hemoglobin ≤ 12.5 g/dL in men and ≤11.5 g/dL in women), (6) excessive alcohol intake (≥60 g of pure alcohol per day, three times the appropriate intake), (7) individuals with irregular lifestyle patterns, including shift workers or night shift workers, (8) known allergies to medications or foods, (9) premenopausal women, (10) anyone deemed unsuitable for participation by the study physician, or (11) individuals who withdrew consent (excluded upon submission of withdrawal form).

The sample size was calculated based on a previous study [5], referencing mean and standard deviation values.

### 2.2. Study Design

This was a randomized, double-blind, placebo-controlled, two-period, two-treatment crossover study. An independent allocation manager, not involved in the study implementation, stratified participants by age and sex and randomly assigned them into the Sequence 1 group or Sequence 2 group. Periods I and II lasted for 2 weeks, separated by a 1-week washout period.

The intervention beverage, MW tea, was prepared from roasted and powdered mulberry leaves and water chestnut husk produced in Kanzaki City, Saga Prefecture. Each 3 g sachet of MW tea contained 2.85 g of mulberry leaves and 0.15 g of water chestnut husks. The active ingredients included 5.10 mg DNJ and 87.3 mg polyphenols. The placebo beverage resembled MW tea in appearance and taste but contained no active components; it was made from corn flour, food colorants, and flavoring agents. Both MW tea and placebo were packaged identically to ensure blinding. Each sachet was mixed with 200 mL of hot water and consumed before meals.

In Period I, the participants in Sequence 1 consumed MW tea before each meal, whereas those in Sequence 2 consumed the placebo. During Period II, the groups switched the assigned beverage. At the beginning and end of each period, medical personnel attached a CGM sensor to the participant’s upper arm. The sensor, which measured interstitial glucose levels with a high correlation to blood glucose, continuously recorded glucose data every 15 min throughout the intervention period [11].

On the first day of each period, the participants arrived at the study site after fasting for at least 12 h (no food intake after 8:00 p.m. on the previous day). After sensor placement and activation, the participants consumed their assigned beverage, followed by a standardized meal (200 g of rice and miso soup containing 7.4 g of carbohydrates). Meal completion time was recorded. For 2 h after the meal, the participants remained at rest while glucose levels were continuously monitored as part of the load test.

Blood samples were collected before and after each intervention period following a fasting period of at least 12 h. Body composition parameters, including weight, body fat percentage, and body mass index (BMI), were assessed using a body composition analyzer (InBody, Tokyo, Japan).

During the intervention period, the participants recorded their beverage intake and adverse symptoms using a daily diary. Physical activity during waking hours was measured using an accelerometer (wGT3X-BT, Acti-Japan Co., Ltd., Tokyo, Japan), and sleep was monitored using a wrist-worn device (ACCELStars Co., Ltd., Kurume, Japan) with sleep–wake state estimation based on the ACCEL algorithm [12].

To assess changes in dietary habits before and during the study, nutrient intake was evaluated using the Brief Diet History Questionnaire (BDHQ) [13,14], which is a simplified version of the self-administered diet history questionnaire.

The study protocol was approved by the Ethics Committee of the Saga University Faculty of Medicine (approval no. R4-10) and was conducted in accordance with the Declaration of Helsinki. The trial was registered in the UMIN Clinical Trials Registry (UMIN000055353). The participants were informed that compensation would be provided only upon full completion of the study schedule.

### 2.3. Outcome Measures

#### 2.3.1. Primary Outcome

The primary outcome of evaluating the efficacy of continuous MW tea intake in suppressing postprandial blood glucose elevation was the coefficient of variation (CV) of glucose levels obtained from the FreeStyle Libre data. CV is a metric commonly used in CGM studies to assess glycemic variability [15].

For the CV calculation, glucose data from the 10 central days of each 14 d intervention period were used, excluding the first and last 2 days, to eliminate the effects of standardized meals and pre-blood-draw fasting. The CV was calculated from the standard deviation and mean of all glucose values recorded for each participant during the 10-day window. Days on which the participants failed to consume the beverage (as documented in the intake diary) were excluded from the analysis.

For safety assessment, the following indicators derived from CGM data were used: (1) the occurrence of hypoglycemia, defined as glucose levels below 70 mg/dL; (2) the duration of hypoglycemia; and (3) the area under the curve (AUC) below the 70 mg/dL threshold, which represents the area between the glucose curve and the 70 mg/dL line (referred to as “70-below AUC”).

#### 2.3.2. Secondary Outcomes

The effect of a single dose of MW tea on postprandial blood glucose levels was evaluated using the AUC of glucose level changes after the consumption of a standardized meal on the first day of each intervention period [15,16]. To standardize the data across participants, the blood glucose level immediately before a meal (self-reported) was set as the baseline (0), and the subsequent incremental changes were used to calculate the AUC.

For a broader assessment of efficacy and safety, various hematological parameters were measured before and after each intervention period. These included white blood cell count, red blood cell count, hemoglobin, hematocrit, mean corpuscular volume, mean corpuscular hemoglobin, mean corpuscular hemoglobin concentration, platelet count; liver and kidney function markers such as aspartate aminotransferase (also known as glutamic–oxaloacetic transaminase), alanine aminotransferase (also known as glutamic–pyruvic transaminase), total bilirubin, creatinine, and uric acid; lipid profile markers including high-density lipoprotein cholesterol, total cholesterol, low-density lipoprotein (LDL) cholesterol, and triglyceride; glucose metabolism markers such as glucose, HbA1c, insulin, glycoalbumin, 1,5-anhydroglucitol (1,5-AG), and C-peptide; the inflammatory marker C-reactive protein (CRP); and the nutritional marker albumin.

The changes before and after each period were compared to assess the physiological effects of MW tea consumption.

#### 2.3.3. Additional Variables

Other potentially relevant variables related to glycemic variability were analyzed as supplementary indicators. Body composition changes in weight, body fat percentage, and BMI were calculated before and after each intervention and control period. Physical activity and sleep were assessed using accelerometer and sleep monitoring data, from which the following averages were calculated for each period: caloric expenditure (kcal/h), metabolic equivalents (METs), and sleep duration. Subjective symptoms were recorded based on participants’ intake diaries, including the frequency of hypoglycemic symptoms (e.g., palpitations, cold sweats, tremors, blurred vision, and drowsiness), gastrointestinal symptoms (e.g., diarrhea, abdominal pain, bloating), and skin symptoms.

### 2.4. Statistical Analysis

Participant background characteristics, age, sex, baseline values of key body measurements, and blood-glucose-related parameters (glucose, insulin, HOMA-IR, C-peptide, glycoalbumin, HbA1c, 1,5-AG, and total cholesterol) are summarized. Continuous variables are expressed as median and IQR, and categorical variables are expressed as counts and percentages.

The primary outcome, the CV of continuous glucose monitoring data, was analyzed using a linear mixed-effects model. Fixed effects included intervention (MW tea or placebo), sequence (1 or 2), and period (I or II), whereas participant ID was treated as a random effect. This model was used to estimate the effect sizes of interventions.

For the safety assessment, the occurrence of hypoglycemia (<70 mg/dL) was compared between the intervention and control periods using McNemar’s test. The duration of hypoglycemia and AUC below 70 mg/dL were compared using the Mann–Whitney U test.

For secondary outcomes, the AUCs of blood glucose levels 1, 2, and 3 h after the standardized meal on the first day of each period were compared between the MW tea and placebo groups using paired *t*-tests.

Changes in blood test values, body measurements, average physical activity, and sleep duration during each period were compared between the intervention and control groups using paired *t*-tests.

Self-reported symptoms recorded in the intake diaries were analyzed using McNemar’s test to compare the frequency of occurrence between the MW tea and placebo periods. A significance level of 5% was used for all statistical tests.

Regarding missing data, the following instances were observed: in AUC, one missing data point was noted for one participant at one time point. In blood tests, there was one missing value for one participant at one time point and one missing item for another participant. In body measurements, there was one missing point for one participant. In physical activity data, there was one missing point for one participant. In sleep monitoring data, there was one missing point for one participant. The missing data were assumed to be completely at random. Therefore, participants were included in each analysis only when the relevant data were available.

All statistical analyses were performed using Python (version 3.11.9) and R (version 4.2.2). The Python libraries used included NumPy, Pandas, and SciPy. The R packages included tidyverse, ggplot2, lme4, lmerTest, and emmeans.

## 3. Results

Among the 34 individuals who were initially allocated to the two groups, all participants gathered at the research site on the first day of the study and received a detailed explanation of the study procedures. Written informed consent was then obtained from each participant prior to the baseline measurement and the start of the intervention. At this stage, three participants withdrew consent and did not proceed with the trial.

**Figure 1:**
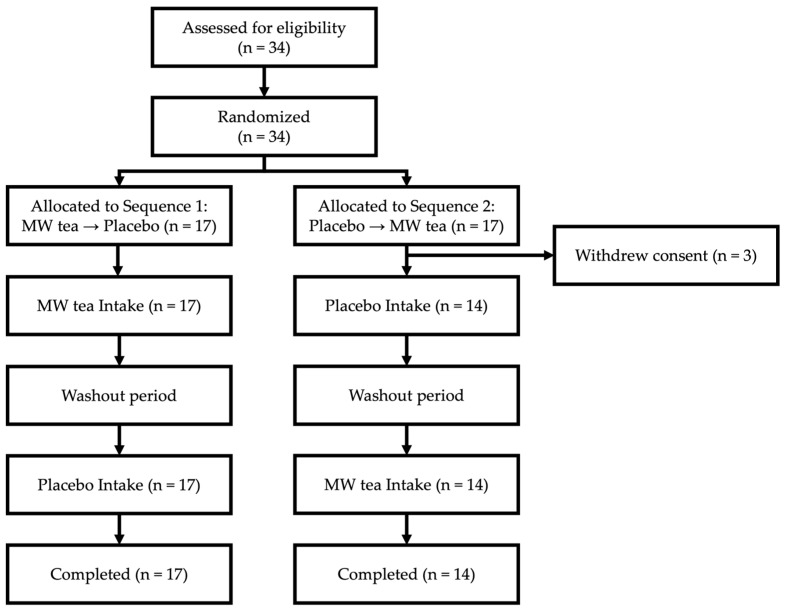
Participant flow diagram.

As a result, a total of 31 participants completed the study schedule (Sequence 1: *n* = 17, Sequence 2: *n* = 14) and were included in the final analysis (Figure 1). The final analysis included 31 participants who completed the study as scheduled, with 17 assigned to Sequence 1 and 14 assigned to Sequence 2 (Figure 1). One participant in Sequence 2 lacked baseline blood test data due to having eaten breakfast before arriving at the study site. The participant met all other eligibility criteria and completed the trial. The baseline characteristics of the study participants are presented in Table 1.

**Figure 2:**
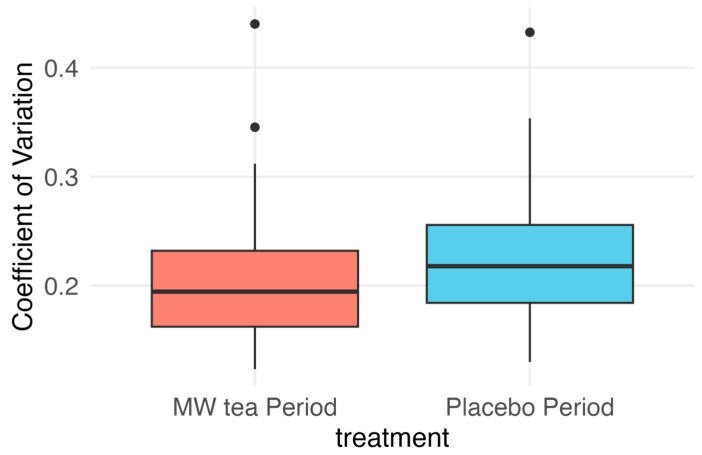
Comparison of coefficient of variation in glucose levels between mw tea and placebo periods in a crossover design.

The primary outcome, the CV of continuous glucose levels during the intervention (MW tea) and control (placebo) periods, is illustrated using boxplots in Figure 2. The mixed-effects model analysis showed that the intervention effect was statistically significant, whereas the sequence and period effects were not. Specifically, after accounting for potential carryover and period effects, the CV during the MW tea period was significantly lower than during the placebo period, with a difference of 0.02 (95% CI: 0.01–0.03, *p* = 0.0006) (Table 2).

**Figure 3:**
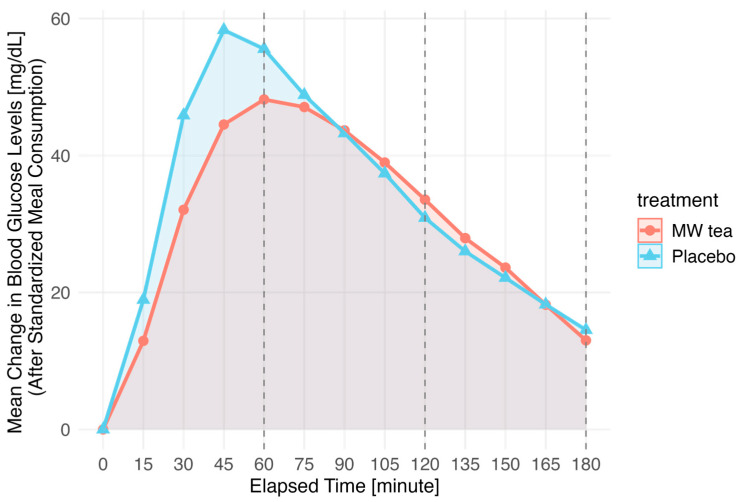
Mean glucose changes over 180 min following standardized meal.

The secondary outcome, the postprandial glucose response following standardized meal intake, is shown in Figure 3 and Table 3. Figure 3 illustrates the time course of postprandial glucose changes and is intended to provide a visual representation of the response curve. Statistical comparisons of the AUC values at 1 h, 2 h, and 3 h are summarized in Table 3. A significant difference was observed in the AUC at 1 h, which was lower in the MW tea group compared to the placebo group (*p* = 0.02). No differences were observed at 2 h and 3 h.

Regarding safety, 18 participants experienced at least one episode of hypoglycemia (<70 mg/dL) during the MW tea period compared to 23 participants during the placebo period. McNemar’s test indicated no statistically significant differences between the groups (*p* = 0.13). The median (IQR) duration of hypoglycemia was 0.25 (0.0–3.5) h in the MW tea period and 1.5 (0.25–6.75) h in the placebo period. The median (IQR) 70-below AUC was 0.5 (0.0–18.9) for MW tea and 9.5 (0.1–69.9) for placebo, with no significant differences observed (*p* = 0.12 and *p* = 0.07, respectively).

Analysis of blood test data revealed that the increase in 1,5-AG levels during the MW tea period was significantly (*p* = 0.005) greater than that during the placebo period. Total cholesterol levels decreased during the MW tea period and increased during the placebo period, resulting in a significant between-group difference (*p* = 0.021) (Table 3).

There were no significant differences in the average calorie expenditure (kcal/h), mean METs, or average sleep duration between the two periods, as measured by the activity tracker and sleep monitor (Table 3). McNemar’s test revealed no significant between-group differences in the self-reported symptoms recorded in the intake diary, including hypoglycemic (*p* = 0.56), gastrointestinal (*p* = 0.10), and skin symptoms (*p* = 1.0).

Nutrient intake results from the BDHQ are shown in Table 4. This questionnaire reflected the one-month periods before and during the trial. Although the total energy intake and food weight showed significant differences, there were no significant differences in protein, fat, or carbohydrate intake.

Comparison of the coefficient of variation (CV) in glucose levels between MW tea and placebo periods in a crossover trial. A mixed-effects model showed a significantly lower CV during the MW tea period than during the placebo period (see Table 2 for statistical results).

This figure shows the mean changes in blood glucose levels over 180 min following the consumption of a standardized meal on the first day of each intervention period. The baseline (0) was defined as the blood glucose level immediately before the meal. The plotted values represent the average glucose changes at each point. Statistical comparisons were conducted only for AUCs at 1 h, 2 h, and 3 h (Table 3), with a significant difference observed at 1 h (*p* < 0.05).

## 4. Discussion

In this study, we conducted a crossover trial to evaluate the effects of Kanzaki MW tea on continuous glycemic control. The primary outcome, the CV of glucose levels measured using CGM, was significantly lower during the MW tea period than during the placebo period (*p* = 0.0006). The mixed-effects model analysis indicated no significant carryover or period effects, and as shown in Table 1, there were no extreme imbalances in baseline characteristics between sequences. Additionally, the physical activity and sleep data did not differ significantly between the MW tea and placebo groups. These results support the efficacy of MW tea in glycemic management.

Even after performing a sensitivity analysis using the Wilcoxon signed-rank test to account for outliers, as illustrated in Figure 2, the CV remained significantly lower during the MW tea period, reinforcing the robustness of the findings.

Regarding the secondary outcome, postprandial blood glucose variation after a standardized meal, a significant reduction was observed in the 1 h AUC but not in the 2 or 3 h AUCs. Figure 3 suggests that the peak glucose level was lower and delayed following MW tea consumption compared with the placebo. These findings suggest that MW tea may primarily suppress early postprandial glucose spikes and that premeal consumption is particularly effective.

Although fasting glucose levels did not show significant changes in blood test analysis, a significant increase in 1,5-AG levels was observed. 1,5-Anhydroglucitol (1,5-AG) is a sugar alcohol that competes with glucose for renal reabsorption and is sometimes used as a short-term marker of glycemic control [17]. When postprandial hyperglycemia persists, glucose saturates renal tubular reabsorption, which, in turn, inhibits 1,5-AG reabsorption and reduces its blood concentration. Therefore, the observed increase in 1,5-AG levels during the MW tea period suggests that MW tea suppresses postprandial hyperglycemia and consequently reduces urinary glucose excretion.

The glycemic control effects of MW tea are likely attributable to its polyphenol and DNJ contents. Polyphenols are known to inhibit α-amylase and α-glucosidase, enzymes responsible for carbohydrate digestion, thereby slowing the breakdown of dietary carbohydrates and mitigating rapid increases in postprandial glucose levels [18,19]. Additionally, polyphenols may enhance insulin sensitivity, supporting efficient glucose uptake by cells [16,17]. Specific polyphenols such as resveratrol and curcumin have been shown to improve insulin resistance and promote glucose metabolism in the liver and skeletal muscles [18,19,20]. Furthermore, they have been reported to reduce inflammation and oxidative stress, which may help prevent the progression of diabetes and insulin resistance. However, in this study, CRP levels, a marker of inflammation, did not decrease with MW tea intake, suggesting that anti-inflammatory effects were not evident. Further investigation is needed to explore this pathway.

DNJ is a potent α-glucosidase inhibitor that reduces postprandial blood glucose levels [21]. In particular, the husk of the water chestnut used in MW tea has been shown to contain hydrolysable polyphenols such as eugeniin, 1,2,3,6-tetra-O-galloyl-D-glucopyranose (TGG), and trapain, which exhibit inhibitory effects on α-amylase and α-glucosidase [5]. These compound-specific mechanisms may complement the known actions of DNJ, a potent α-glucosidase inhibitor that reduces postprandial blood glucose levels [5]. Previous randomized controlled trials using DNJ-rich mulberry leaf powder have also demonstrated significant reductions in postprandial glucose and insulin secretion [6,7]. Moreover, the combination of mulberry leaves and water chestnut husks has been shown to synergistically inhibit carbohydrate-digesting enzymes [5]. While direct comparisons with earlier studies are limited due to differences in test meals and measurement methods, our findings suggest that the combined use of mulberry leaves and water chestnut husks may produce a synergistic effect, not only lowering the peak postprandial glucose level but also delaying its occurrence.

Regarding safety, no significant differences were observed between the MW tea and placebo in terms of hypoglycemia occurrence (<70 mg/dL), AUC < 70 mg/dL, or self-reported hypoglycemic, gastrointestinal, or skin symptoms. Blood tests revealed no significant changes in hematological, liver, renal, or inflammatory markers, suggesting that short-term MW tea intake is safe. Unlike previous studies that relied primarily on self-reporting of adverse events, this study incorporated objective measures, such as CGM and blood analysis, alongside intake diaries to enhance the reliability of safety assessments.

Additionally, total cholesterol significantly decreased after MW tea intake, whereas LDL cholesterol also showed a decreasing trend, although this was not statistically significant. Polyphenols and DNJ suppress cholesterol absorption via various mechanisms. For instance, polyphenols directly bind to the cholesterol transporter Niemann-Pick C1-like 1, downregulating its expression and inhibiting cholesterol absorption. Some polyphenols also increase *CYP7A1* expression, reduce intestinal bile acid transporters, promote bile acid excretion, and alter the gut microbiota composition [22,23]. DNJ and DNJ-rich mulberry extracts reportedly enhance adiponectin mRNA expression in white adipose tissue, increase plasma adiponectin levels, activate *AMPK* mRNA expression, reduce plasma triacylglycerol levels, and decrease visceral fat mass and adipocyte size [24]. These mechanisms suggest that MW tea may have beneficial effects not only on glycemic control but also on lipid metabolism.

Although the BDHQ dietary survey could not directly compare diet content between the intervention and control periods (owing to the one-month recall window), it was used to assess whether the participants maintained consistent dietary patterns throughout the study. No significant differences were found in protein, fat, or carbohydrate intake, although both energy and food weights decreased slightly during the study period. These changes could potentially influence the glucose measurements. However, body composition measurements revealed no significant differences between the periods, and body weight and BMI decreased slightly in both periods. As the study was blinded, it is unlikely that the participants intentionally reduced their calorie intake in only one period. Therefore, the observed effectiveness of postprandial glycemic control is unlikely to be confounded by changes in dietary intake.

This study has some limitations. The intervention period was relatively short, and the sample size was limited. Further research is required to investigate the long-term effects and generalizability of MW tea’s efficacy and safety of MW tea in a broader population. In addition, several secondary outcomes were evaluated in this study. Each outcome was based on a distinct null hypothesis and analyzed independently; therefore, we did not perform a statistical correction for multiple comparisons. However, we recognize that if the null hypotheses were not considered independent, the analysis should account for the increased risk of type I error. The results should thus be interpreted with appropriate caution.

Despite these limitations, this study assessed the effects of 2-week MW tea intake using a randomized, double-blind, placebo-controlled, two-period crossover design, which minimized inter-individual variation. Comprehensive evaluation using CGM, blood tests, physical activity monitoring, sleep tracking, and dietary assessment allowed for a rigorous assessment while minimizing bias. These findings provide high-level evidence supporting the efficacy and safety of short-term MW tea consumption for suppressing postprandial blood glucose elevation.

## 5. Conclusions

This randomized, double-blind, placebo-controlled crossover study evaluated the postprandial-blood-glucose-suppressing effect and safety of a 2-week intake of Kanzaki MW tea, which contains 2.85 g of mulberry (*Morus alba*) leaves and 0.15 g of water chestnut (*Trapa japonica*) husks per serving. The primary outcome, the CV of glucose levels, significantly reduced during the MW tea period, indicating that short-term MW tea consumption effectively suppressed postprandial blood glucose elevation.

No significant differences were observed between the MW tea and placebo groups in the occurrence of hypoglycemia, hepatic or renal function markers in blood tests, inflammatory markers, or self-reported gastrointestinal and skin symptoms. These results support the safety of consuming MW tea.

These findings demonstrate a postprandial-blood-glucose-suppressing effect in healthy individuals; however, evaluation in populations at risk for—or living with—diabetes and in longer trials is required before clinical recommendations can be made.

## Figures and Tables

**Table 1:** Baseline characteristics of study participants by sequence. (Values are presented as Median (IQR) or *n* (%)).

	Overall (*n* = 31)	Sequence 1 (*n* = 17)	Sequence 2 (*n* = 14)
**Age**	56 (48.5–65.5)	57 (48.0–66.0)	54.5 (49.2–64.8)
**Gender**			
Male	13 (23%)	7 (12%)	6 (11%)
Female	18 (32%)	10 (18%)	8 (15%)
**Body Measurements**			
Height [cm]	161.6 (155.9–170.0)	161.6 (156.6–170.5)	162.1 (155.3–168.7)
Weight [kg]	63 (51.6–70.2)	66 (53.8–75.2)	55.2 (49.5–66.3)
Body Fat [%]	26 (23.2–31.7)	27.8 (24.3–33.3)	24.7 (21.2–28.0)
BMI	23.3 (20.2–25.0)	24.2 (21.4–27.6)	21.4 (19.9–24.0)
**Blood Test Data**	(*n* = 30)	(*n* = 17)	(*n* = 13)
Glucose [mg/dL]	101 (95.2–113.2)	104 (97.0–107.0)	97 (92.0–115.0)
Insulin [μIU/mL]	6.2 (3.8–8.8)	7.3 (5.2–8.6)	5.5 (3.7–8.9)
HOMA-IR [mg/dL·μIU/mL]	1.6 (0.9–2.4)	1.7 (1.2–2.3)	1.3 (0.8–2.5)
C-Peptide [ng/mL]	1.5 (1.0–2.0)	1.5 (1.4–1.9)	1.2 (0.9–2.0)
Glycoalbumin [%]	14.1 (13.2–14.8)	13.8 (13.0–14.6)	14.2 (13.5–15.1)
HbA1c (NGSP) [%]	5.5 (5.3–5.7)	5.5 (5.4–5.7)	5.4 (5.3–5.6)
1,5-AG [μg/mL]	19.4 (14.1–24.3)	20.3 (15.1–27.9)	18.4 (13.9–24.1)
T-Cholesterol [mg/dL]	227.5 (196.0–247.0)	226 (195.0–266.0)	229 (199.0–243.0)

BMI—Body Mass Index; HbA1c—Glycated Hemoglobin; 1,5-AG—1,5-Anhydroglucitol; HOMA-IR—Homeostasis Model Assessment of Insulin Resistance.

**Table 2:** Results of mixed-effects model analysis for CV of glucose levels.

	Period 1	Period 2	Within Subject Difference (MW Tea—Placebo)
**Sequence 1: MW tea → Placebo**			
Mean (SD)	0.21 (0.07)	0.24 (0.07)	−0.025 (0.03)
*n*	17	17	17
**Sequence 2: Placebo → MW tea**			
Mean (SD)	0.22 (0.05)	0.20 (0.07)	−0.016 (0.03)
*n*	14	14	14
**Treatment effect**			
LSMean (95% CI)			−0.0203 (−0.031 to −0.010)
*n*			31
Paired Analysis			*p* = 0.0006

CV—Coefficient of Variation; MW tea—Mulberry Leaf and Water Chestnut Tea.

**Table 3:** Changes in blood parameters, body composition, activity, sleep, and self-reported symptoms between MW tea and placebo periods (Values are expressed as Mean (SD) or Mean Change (Post–Pre) (SD).).

Test Item	MW Tea	Placebo Tea	*p*-Value
**Glucose Levels After Test Meal Consumption (Mean)**			
AUC 1 h	1704 (1145)	2263 (1289)	0.020 *
AUC 2 h	4262 (2502)	4853 (3524)	0.267
AUC 3 h	5658 (3280)	6190 (4679)	0.484
**Blood Test Data (Mean Change ^†^)**			
**Peripheral Blood General Test**			
White Blood Cell [μL]	183.333 (925.532)	30.000 (740.992)	0.462
Red Blood Cell [×10^4^/μL]	2.067 (17.767)	7.833 (17.313)	0.245
Hemoglobin [g/dL]	0.030 (0.565)	0.247 (0.483)	0.164
Hematocrit [%]	0.190 (1.791)	0.710 (1.452)	0.231
MCV [fL]	−0.010 (1.196)	−0.047 (1.124)	0.913
MCH [pg]	−0.057 (0.409)	0.010 (0.330)	0.494
MCHC [%]	−0.080 (0.506)	0.040 (0.425)	0.361
Platelet Count [×10^4^/μL]	0.113 (1.954)	0.227 (2.132)	0.864
**Liver Function**			
AST (GOT) [U/L]	−0.067 (3.571)	0.133 (3.785)	0.827
ALT (GPT) [U/L]	0.733 (6.125)	−0.667 (4.671)	0.228
Total Bilirubin [mg/dL]	0.017 (0.207)	0.003 (0.154)	0.791
**Renal Function**			
Creatinine [mg/dL]	−0.004 (0.044)	−0.005 (0.048)	0.958
Uric Acid (UA) [mg/dL]	0.000 (0.538)	−0.013 (0.452)	0.919
**Lipid Metabolism**			
HDL Cholesterol [mg/dL]	−3.267 (5.723)	−0.100 (4.950)	0.053
LDL Cholesterol [mg/dL]	−7.400 (18.350)	0.633 (11.987)	0.076
Total Cholesterol [mg/dL]	−7.767 (19.224)	6.167 (18.424)	0.021 *
Triglycerides [mg/dL]	7.167 (71.705)	28.133 (64.959)	0.325
**Glucose Metabolism**			
Glucose [mg/dL]	−1.233 (7.632)	0.200 (7.073)	0.523
HbA1c (NGSP) [%]	−0.030 (0.134)	−0.020 (0.103)	0.794
Insulin [μIU/mL]	0.843 (3.545)	0.308 (3.609)	0.580
Glycoalbumin [%]	0.143 (0.673)	0.217 (0.703)	0.635
1,5-AG [μg/mL]	0.937 (1.234)	0.037 (1.100)	0.005 *
**Glucose Metabolism**			
Albumin (Alb) [g/dL]	0.043 (0.267)	0.167 (0.199)	0.070
C-Peptide (CPR) [ng/mL]	0.078 (0.558)	0.063 (0.410)	0.914
C-Reactive Protein (CRP) [mg/dL]	0.016 (0.225)	0.018 (0.177)	0.958
**Other Markers**			
Albumin (Alb) [g/dL]	0.043 (0.267)	0.167 (0.199)	0.070
C-Peptide (CPR) [ng/mL]	0.078 (0.558)	0.063 (0.410)	0.914
C-Reactive Protein (CRP) [mg/dL]	0.016 (0.225)	0.018 (0.177)	0.958
**Body Measurements (Mean Change ^†^)**			
Weight [kg]	−0.223 (0.67)	−0.253 (0.40)	0.834
Body Fat Percentage [%]	0.293 (1.03)	0.210 (1.18)	0.772
BMI [kg/m^2^]	−0.083 (0.26)	−0.097 (0.15)	0.810
**Activity and Sleep Monitors (Mean)**			
Caloric Expenditure [kcal/h]	12.16 (6.23)	13.13 (6.33)	0.184
Physical Activity Intensity [1/h]	1.74 (0.18)	1.76 (0.19)	0.341
Daily Sleep Duration [h]	5.72 (1.13)	5.78 (1.04)	0.605
Nocturnal Awakenings [h]	0.74 (0.48)	0.77 (0.46)	0.619
**Self-Reported Symptoms (Count)**			
Hypoglycemic Symptoms	1	2	0.56
Gastrointestinal Symptoms	6	1	0.10
Dermatological Symptoms	1	1	1.0

* *p* < 0.05. Statistical comparisons were performed using paired *t*-test for all variables except self-reported symptoms, which were analyzed using McNemar’s test. ^†^ Mean Change calculated as postintervention minus preintervention. AST (GOT)—Aspartate Aminotransferase (Glutamate Oxaloacetate Transaminase); ALT (GPT)—Alanine Aminotransferase (Glutamate Pyruvate Transaminase); HDL—High-Density Lipoprotein; LDL—Low-Density Lipoprotein; HbA1c—Glycated Hemoglobin; 1,5-AG—1,5-Anhydroglucitol; CRP—C-Reactive Protein; BMI—Body Mass Index.

**Table 4:** BDHQ nutritional analysis: comparison between preintervention and during-intervention periods.

Item	Precrossover Period Mean (SD)	During-Crossover Period Mean (SD)	*p*-Value
Estimated Energy Requirement [kcal/day]	2189.3 (307.0)	2188.6 (307.3)	0.160
Energy [kcal/day]	1839.1 (530.1)	1696.1 (466.7)	0.015 *
Weight [g/day]	2139.5 (683.0)	1974.8 (621.0)	0.040 *
Water [g/day]	1743.8 (597.7)	1611.6 (536.0)	0.061
Protein [g/day]	65.6 (17.9)	63.7 (18.7)	0.411
Fat [g/day]	54.0 (15.3)	53.0 (15.3)	0.657
Carbohydrates [g/day]	246.5 (97.0)	220.3 (78.6)	0.067

Values are expressed as mean (standard deviation). The data represent estimated nutrient intake during the precrossover period and during the crossover period, based on responses to the BDHQ. BDHQ—Brief Diet History Questionnaire. * *p* < 0.05, based on paired *t*-test.

## Data Availability

The datasets used in this study are available from the corresponding author upon reasonable request. The data are not publicly available due to privacy concerns.

## Abbreviations

The following abbreviations are used in this manuscript:

1,5-AG	1,5-Anhydroglucitol
ACCEL	Accelerometer algorithm (specific algorithm name, not a broader abbreviation)
ALT (GPT)	Alanine aminotransferase (glutamate pyruvate transaminase)
AMPK	AMP-activated protein kinase
AST (GOT)	Aspartate aminotransferase (glutamate oxaloacetate transaminase)
AUC	Area under the curve
BDHQ	Brief Diet History Questionnaire
BMI	Body mass index
CGM	Continuous glucose monitoring
CI	Confidence interval
CRP	C-Reactive protein
CV	Coefficient of variation
DNJ	1-Deoxynojirimycin
GPT	Glutamate pyruvate transaminase (see ALT)
GOT	Glutamate oxaloacetate transaminase (see AST)
HbA1c	Glycated hemoglobin
HOMA-IR	Homeostasis model assessment of insulin resistance
LDL	Low-density lipoprotein
METs	Metabolic equivalents
MW tea	Mulberry leaf and water chestnut tea
UMIN	University Hospital Medical Information Network
